# Infrared Ion Spectroscopy Combined with Ion Mobility Spectrometry for Identification of Caffeine Metabolite Isomers and Protomers

**DOI:** 10.1021/jasms.5c00356

**Published:** 2026-01-28

**Authors:** Gustavo Cervi, Thiago C. Correra

**Affiliations:** Department of Fundamental Chemistry, 153989 Institute of Chemistry, University of São Paulo, Av. Prof. Lineu Prestes, 748, Cidade Universitária, São Paulo, São Paulo 05508-000, Brazil

**Keywords:** caffeine, metabolites, ion spectroscopy, ion mobility, mass spectrometry

## Abstract

Chromatography combined with tandem mass spectrometry is a conventional strategy for metabolite differentiation and identification. However, coelution and overlapping fragmentation patterns often limit confident assignment of isomeric and isobaric species. Caffeine metabolites represent a particularly challenging case. Here, we demonstrate a high-field asymmetric waveform ion mobility spectrometry (FAIMS) approach coupled with infrared multiple photon dissociation (IRMPD) ion spectroscopy for the identification of the major caffeine metabolites paraxanthine (PX), theobromine (TB), and theophylline (TP). FAIMS provided baseline separation of these isomers into four distinct populations, including two distinct protomers of protonated TB. The nature of each FAIMS population was probed by IRMPD, which enabled structural assignment and revealed unique protomeric signatures for PX and TP. This combined FAIMS–IRMPD workflow not only resolves isomeric metabolites but also distinguishes protomeric and tautomeric forms, expanding the scope of ion mobility–spectroscopy approaches in metabolite analysis.

## Graphical abstract

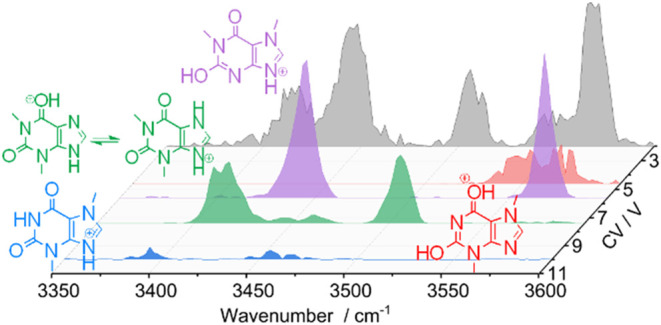

## Introduction

The correct identification of isomeric molecules is relevant in many areas of knowledge, especially in biological contexts, where it may provide insights into the overall biochemical state of an organism, with implications for the diagnostics and treatment of a series of diseases.^1–3^

Chromatography techniques coupled to mass spectrometry are typically the methods of choice for analyzing complex matrices, as the combination of chromatographic separation and tandem MS can often differentiate isomers, depending on their chemical characteristics and fragmentation pathways.^4–10^ Nevertheless, these approaches rely on the availability of reference standards so that chromatographic behavior and individual fragmentation patterns of each isomer can be established in advance.^11–15^ Consequently, they are not suitable for all classes of analytes, particularly for species that cannot be isolated as standards or that may not withstand conventional chromatographic conditions, such as short-lived or chemically unstable analytes.^16–20^ These considerations highlight the importance of developing alternative gas-phase strategies capable of distinguishing isomeric ions without relying on chromatography or isolated analytical standards.

Major caffeine metabolites (Figure 1), including theobromine (TB), theophylline (TP), and paraxanthine (PX), represent a well-studied model system for exploring such gas-phase approaches. Although their analytical standards are readily available and their characterization has been widely reported in various matrices,^2,21–25^ distinguishing these isomeric metabolites rapidly and unambiguously can still be nontrivial depending on the methods employed. Their accurate identification remains relevant, particularly because the distribution of caffeine metabolites is frequently used as an indicator of human-derived contamination in wastewater.^26–30^

**Figure 1:**
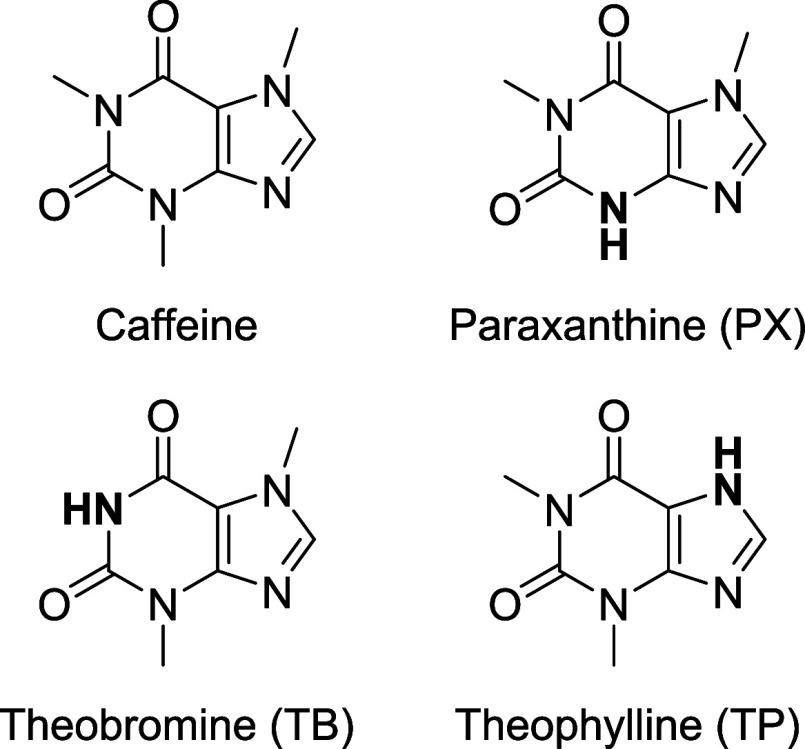
Caffeine and its three major metabolites: paraxanthine (PX), theobromine (TB), and theophylline (TP).

For instance, the difficulty in resolving PX and TP was reported to render slow chromatographic methods or to require complex sample preparation, as reported by Campíns-Falcó and co-workers and Aresta and co-workers.^22,23^ In this context, Choi and co-workers presented,^31^ in 2013, a chromatographic method for the fast evaluation of caffeine and its metabolites using an isocratic 0.2% formic acid in distilled water mobile phase in 7 min using acetaminophen as an internal standard. In 2024, Goldner and co-workers^32^ revisited this topic and developed a green reversed-phase liquid chromatography method to determine caffeine and its major metabolites in diverse matrices using 6 to 10% ethanol in water as the mobile phase in less than 6 min. Despite their success, the pair PX/TP still proved challenging and showed lower separation resolution between the metabolites evaluated.

Ion spectroscopy and ion mobility techniques can be coupled to mass spectrometry to allow the identification of many species of interest,^33–37^ including caffeine and its metabolites.^38–40^ Van Outersterp and co-workers, in 2019, showcased the relevance of infrared ion spectroscopy (IRIS) for metabolite analysis.^41^ To that end, HPLC-MS separated fractions containing the target metabolites were evaluated by IRIS at the fingerprint region, allowing the infrared spectra of the targeted species to be acquired and compared with quantum-chemical predicted absorption spectra of putative targets selected from a metabolite database.

This methodology was shown to be effective in the reference-standard free metabolite identification and was later expanded to allow online IRIS during HPLC separation using heart-cutting liquid chromatography.^42^ Despite their success, the use of previous chromatographic techniques may still prevent unresolved and transient species from being evaluated.

Recently, Geue and co-workers employed helium nanodroplet IR spectroscopy in the fingerprint region, without prior chromatographic separation, to characterize and distinguish the protomeric and tautomeric forms of caffeine and its metabolites.^43^ Their work clearly illustrates the analytical importance of identifying protomers and tautomers in the gas-phase, as the three major metabolites showed spectral signatures consistent with contributions from multiple protomeric/tautomeric structures based on comparison with DFT-predicted bands. These findings emphasize the value of methodologies capable of separating ion populations prior to infrared characterization and suggest that integrating gas-phase separation with IR spectroscopy may enable more definitive structural differentiation.

Ion mobility spectrometry (IMS) approaches can be used to enhance or even to replace chromatographic steps when coupled to mass spectrometry.^44–47^ In 2022, Sepman and co-workers^48^ investigated the gas-phase populations of caffeine metabolites using traveling wave cyclic ion mobility spectrometry (TWIMS). Among the compounds studied, protonated PX consistently exhibited two distinct drift time peaks centered at approximately 62.5 and 66.2 ms. These peaks correspond to gas-phase protomers/tautomers, and their relative abundances remained similar across solvents, with the lower-drift-time species being more abundant. Protonated TP, by contrast, displayed a single peak around 64.2 ms, indicating a single predominant gas-phase structure. Notably, protonated TB also presented two well-resolved peaks, both in water and acetonitrile, with one of its protomeric forms exhibiting a drift time near 64.5 ms—overlapping significantly with the arrival time of protonated TP. Despite the characterization of the protomers/tautomers achieved on that study, the similar arrival times would hinder confident differentiation of these isomers in a mixture, particularly in untargeted analyses, showing that the ability to resolve and probe the diverse isomers in the gas-phase is the limiting factor in this scenario, fostering the development of more capable IMS techniques coupled with diverse ion fragmentation schemes.^49^

Differently from the other ion mobility variants, High-Field Asymmetric Waveform Ion Mobility Spectrometry (FAIMS) separates isomers based on their differential ion mobility in the high- and low-electric-field regimes produced by an asymmetric waveform.^50^ The interaction of the ions with the buffer gas in the presence of this oscillating electric field affects the ion dispersion in the ion mobility cell and is dependent on the differential mobility parameters of the ions as determined by their interactions with the separation gas. The mobility parameters can be evaluated by scanning a compensation voltage (CV) that refocuses the ions to the detector. A plot of ion intensity as a function of the compensation voltage produces an IMS spectrum, or ionogram.^51^

This approach is particularly relevant for small polar molecules and metabolites that may not exhibit significant differences in collisional cross section yet display markedly distinct ion clustering and, consequently, differential ion mobility behaviors. Therefore, by coupling FAIMS with infrared spectroscopy, as demonstrated by Berthias and co-workers in 2018 for sarcosine isomers,^52^ mixtures of metabolites can be separated and subsequently identified.

In this context, this work uses FAIMS as a fast alternative to achieve the inline separation of isomeric species of caffeine metabolites and their direct identification by Infrared Multiple Photon Dissociation (IRMPD) spectroscopy, supported by infrared absorption spectra simulated at the B3LYP/aug-cc-pVDZ level.

## Results and Discussion

### FAIMS Results

To evaluate the major caffeine metabolites, we started by acquiring the FAIMS spectrum of a mixture of PX, TP, and TB, as depicted in Figure 2a.

**Figure 2:**
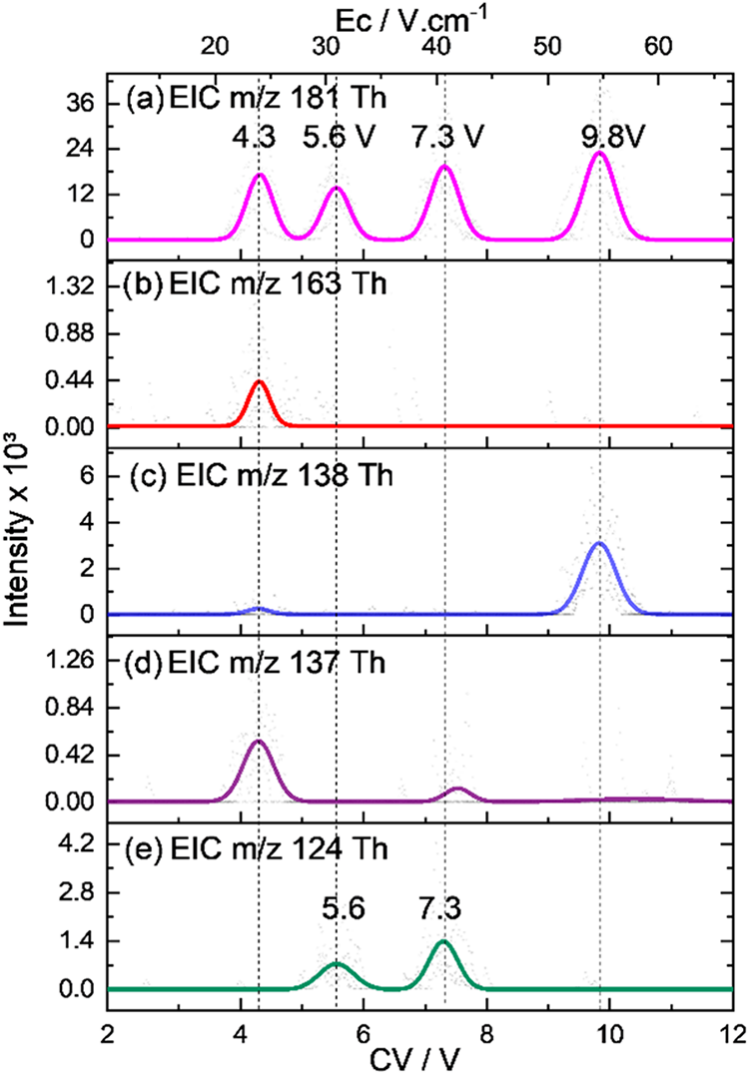
FAIMS spectrum for a mixture containing the three major caffeine metabolites and fragments generated by collision-induced dissociation (CID) at 0.27 V of collision energy for each population shown as extracted intensities of (a) the protonated caffeine metabolites with *m*/*z* 181 and their fragments with *m*/*z* (b) 163, (c) 138, (d) 137, and (e) 124. The FAIMS spectrum of the isolated analytical standards of the metabolites is shown in Figure S1.

This analysis shows that there are 4 baseline resolved populations detected at CV of 4.3, 5.6, 7.3, and 9.8 V for the ion with *m*/*z* 181 correlated to the protonated metabolites. This initial result suggests that at least one of the analytes is detected as more than one population, consistent with the presence of different protomers or tautomers as previously reported by Sepman and co-workers.^48^

Despite the great separation achieved, no identification can be derived directly from this result; therefore, the population-specific collision-induced dissociation (CID) data were obtained by monitoring ion dissociation from the *m*/*z* 181 precursor during the FAIMS separation (Figure 2b–e). The first population at CV 4.3 V dissociates forming fragments with *m*/*z* 137 (loss of 44 Da), 163 (loss of 18 Da), and a minor amount of the fragment with *m*/*z* 138 (loss of 43 Da) (Figure 2). Based on previous reports,^4,48^ these fragments were assigned to the loss of acetaldehyde, water, and isocyanic acid, respectively. The fragment with *m*/*z* 138 was observed only in significant intensities for the population detected at CV 9.8 V, being the only fragment observed for this population. The populations at CV 5.6 and 7.3 V fragment into ions with *m*/*z* 124 (loss of 57 Da), corresponding to the loss of methyl isocyanate. The population at 7.3 V also shows a minor formation of the fragment with *m*/*z* 137 (loss of 44 Da) (Figure 2). Other than the ion with *m*/*z* 163, no other fragment is exclusive to one population in this FAIMS-MS/MS analysis, hindering their structural characterization based solely on their fragmentation pattern.

The analysis of the isolated metabolite standards (Figure S1) was carried out to validate the identification of the populations without the use of analytical standards and showed that the populations at 4.3 and 9.8 V are observed for the protonated TB, confirming that this species is present as two distinct protomer/tautomers, while the populations at 5.6 and 7.3 are observed for PX and TP, respectively. The CID spectra of the three isolated metabolites (Figure 3a–c) show that although distinct, with PX presenting fragments at *m*/*z* 124 and 142, TP yielding mainly *m*/*z* 137 and 124, and TB generating fragments at *m*/*z* 137, 138, and 163 in different proportions, they cannot be used for the unambiguous differentiation of these isomeric metabolites.

**Figure 3:**
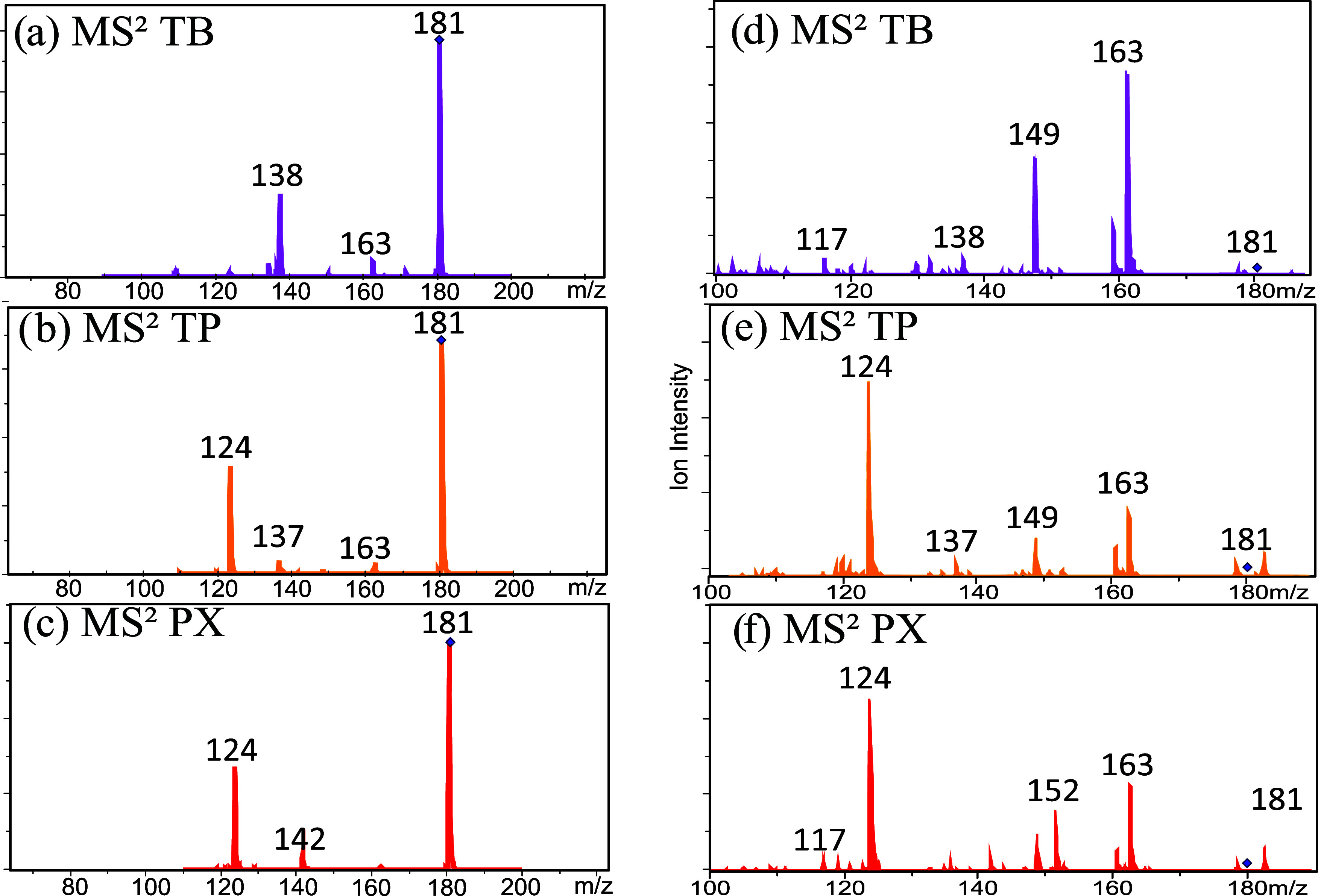
CID spectra of caffeine metabolites under low (a, b, and c) (0.32 V) and high (d, e, and f) (0.41 V) collision energy conditions.

This is particularly true for the TP/PX pair, as both predominantly fragment into the ion with *m*/*z* 124, and their differentiation would depend only on the presence of low-intensity diagnostic ions, as the fragments with *m*/*z* 152, 149, and 142 formed under higher collision energy conditions (Figure 3d–f). Nevertheless, this analysis is not sufficient for the complete characterization of a mixture of these metabolites.

The two distinct populations observed for TB at CV 4.3 and 9.8 V could, in principle, generate different CID patterns, suggesting that the protonation site and tautomeric equilibria play a significant role in the gas-phase behavior. Nevertheless, the CID data obtained by MS/MS without previous IMS separation would not allow this behavior to be evaluated, requiring another technique for fully evaluating the protonation site.

### Protomers and Their Contribution

The IRMPD spectra of the metabolite mixtures (Figure 4a, red trace) and the selected populations (Figure 4b–c) were acquired, and the results were compared to absorption spectra simulated at the B3LYP/aug-cc-pVDZ level of theory (Figure 4, vertical lines). The suitability of this level of theory is discussed elsewhere.^53^

**Figure 4:**
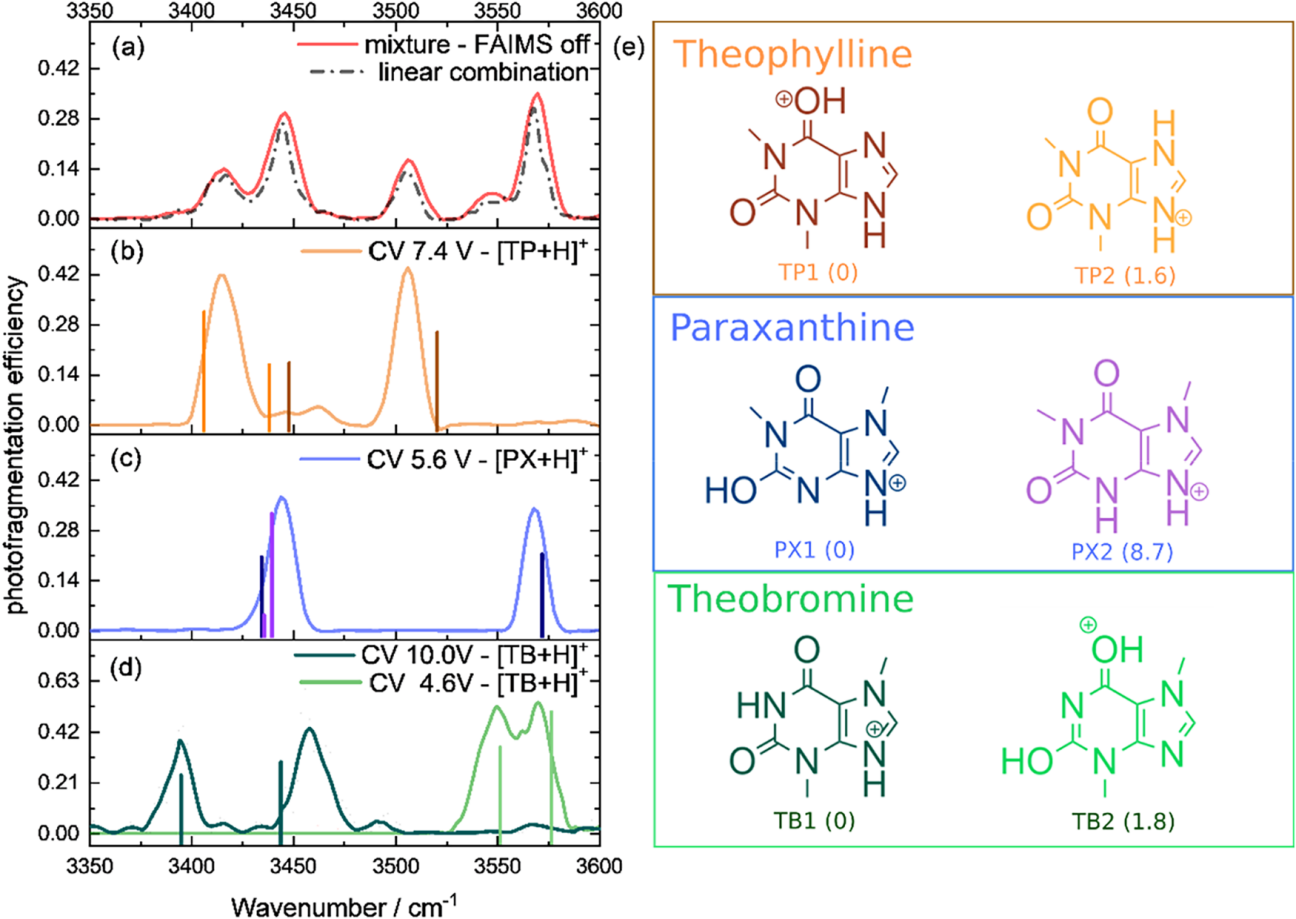
IRMPD spectrum of (a) a mixture of caffeine metabolites and (b–d) for selected FAIMS population. Vertical lines show the absorption bands calculated at the B3LYP/aug-cc-pVDZ level of theory, color coded as depicted in the putative protomers for the caffeine metabolites shown in (e). A 0.9542 scale factor was applied to the calculated frequencies. Values in parentheses indicate the energy in kJ mol^–1^ relative to the lowest energy species. Protomers with relative energy higher than 9 kJ mol^–1^ are not shown, as their vibrational spectra did not contribute to the present discussion.

Figure 4c shows the IRMPD spectra of the population at CV 5.6 V. This vibrational spectrum shows two bands centered at 3444 and 3569 cm^–1^ that could be assigned to the NH^+^ stretch and the free OH stretch of the lowest energy N-protonated iminol form of PX (PX1) based on the absorption bands calculated at 3435 and 3573 cm^–1^, respectively (Figure 4c, vertical lines), as presented in Table 1. The second lowest energy protomer is the amide form of the N-protonated PX (PX2), and its predicted vibrational spectrum shows the symmetric and antisymmetric NH stretches as an envelope at 3436–3440 cm^–1^. Based on the higher energy of this protomer PX2 (8.7 kJ mol^–1^), its contribution was considered negligible, although a mixture of PX1 and PX2 cannot be disregarded based on the IRMPD spectra and the MS^2^ results as both protomers could lose methyl isocyanate to form the fragment with *m*/*z* 124 and show calculated absorption that could be assigned to the experimental band at 3444 cm^–1^. The presence of PX1 and PX2 is also supported by previous reports from Geue and co-workers^43^ and Sepman^48^ and co-workers, using cryogenic IR spectroscopy and cyclic IMS, respectively.

**Table 1:** Comparison of Experimental and Theoretical IRMPD Band Centroids for Each Identified Protomer of Caffeine Metabolites

species	assignment	ν̃_exp_/cm^–1^	ν̃_theor_/cm^–1^	Δν̃/cm^–1^
**TB1**	N–H amide_antisym_ ^a^	3395	3395	0
	N–H imine_sym_ ^a^	3458	3444	13.78
	**RMSD** ^b^			**9.75**
**TB2**	O–H^+^	3549	3551	2.24
	O–H_enol_	3571	3576	5.39
	**RMSD** ^b^			**4.14**
**PX1**	N–H	3444	3435	9.22
	O–H	3569	3573	3.87
	**RMSD** ^b^			**7.01**
**PX2**	N–H amide_antisym_ ^a^	3440	3436	4.12
	N–H imine_sym_ ^a^	3448	3440	8.37
	**RMSD** ^b^			**6.59**
**TP1**	N–H	3463	3448	14.70
	O–H	3506	3521	14.70
	**RMSD** ^b^			**14.69**
**TP2**	N–H_antisym_ ^a^	3415	3407	8.43
	N–H_sym_ ^a^	3446	3439	7.42
	**RMSD** ^b^			**7.93**

a Despite both symmetric and antisymmetric motions being involved in these predicted vibrations, the notations used denote the most active stretch associated with the vibrational frequency.

b RMSD values are calculated as the square root of the mean of the squared experimental and theory frequency differences calculated at the B3LYP/aug-cc-pVDZ level of theory. A 0.9542 scale factor was applied to the calculated frequencies.

The population at CV 7.4 V (Figure 4b) shows two major IRMPD bands at 3415 and 3506 cm^–1^, and some other minor bands between 3446 and 3463 cm^–1^. The band at 3415 cm^–1^ was assigned to the NH stretch calculated at 3407 cm^–1^ for the second lowest energy protomer TP2 (1.8 kJ mol^–1^) in the protonated N amide form (Table 1). The simulated absorption spectrum of this protomer also shows the protonated N stretch at 3439 cm^–1^, which could arguably be correlated to one of the minor IR absorptions observed in the experimental spectrum at 3446 cm^–1^.

The other major band at 3506 cm^–1^ was assigned to the lowest energy O-protonated iminol protomer TP1 that showed a calculated OH stretch at 3521 cm^–1^. An NH stretch was also calculated at 3448 cm^–1^, corresponding to the second minor absorption detected at 3463 cm^–1^. It is interesting to point out that in this case, the experimental OH band is red-shifted from the calculated value, suggesting that some sharing of the proton between both protonation sites may be present.

The presence of TP1 and TP2 is in accordance with previous studies on the infrared spectrum of protonated TP in the fingerprint range.^40,43^ Marta and co-workers^40^ report spectral features consistent with the presence of a protonated carbonyl group relative to TP1 and two N–H wag modes, assigned to the N-protonated protomer TP2, similarly to Geue and co-workers’ results.^43^ Conversely, TP2 was not observed by the previously conducted IMS analysis by Sepman and co-workers.^48^

The populations observed for TB at CV = 4.6 and 10 V show completely different vibrational features. The population at CV 4.6 V shows two unresolved bands at the 3549 and 3571 cm^–1^ range, while the population at CV 10 V shows two distinct bands at 3395 and 3458 cm^–1^. The unresolved bands at the lower CV population were assigned as the protonated carbonyl oxygen, and the OH stretches from the second lowest energy (1.8 kJ mol^–1^) O-protonated iminol form of TB (TB2) predicted at 3551 and 3576 cm^–1^ (Table 1). TB isomers evaluated show relative energies in agreement with a previous report from Sepman and co-workers that showed that the TB2 population interconverted to TB1 during a second IMS separation cycle, suggesting a lower energy for TB1.^48^ The other bands were assigned as the protonated nitrogen NH^+^ stretch and the amide NH stretch of the lowest energy N-protonated amide form TB1, calculated at 3395 and 3444 cm^–1^ and observed at 3395 and 3458 cm^–1^, respectively (Table 1). While Geue and co-workers^43^ reported IR spectra containing contributions from mixed protomeric/tautomeric forms, our data resolve the IR absorptions of each of the two mobility-separated populations, in accordance with previous studies.^40,43^

The IRMPD spectra of the mixture of metabolites (Figure 4a) without FAIMS selection show 5 absorption bands that can be correlated to the individual vibrational features of the individual protomers observed. A linear combination fit was used to the mix the population selected spectra, generating a combination spectrum (Figure 4a, dashed line) that presented a good agreement to the IRMPD spectrum of the mixture of metabolites, with a R^2^ of 0.97964 and absorption bands centered at 3414, 3445, 3506, 3545, and 3566 cm^–1^, in close agreement with the IRMPD spectrum of the isolated species. The linear coefficients found were 0.10 and 0.04 for TB1 and TB2, 0.58 for PX, and 0.28 for TP. The lower contribution of the band assigned to the protonated TB population at 4.6 V (TB2) is expected once its photofragmentation was shown to be lower in comparison to the other species, as it required 25 pulses to achieve a similar photofragmentation efficiency as the species on CV 10 V (TB1), which required 13 laser pulses.

As can be seen in Figure 4, while the MS/MS results show some similar fragmentation patterns that hinder the direct metabolite identification, the FAIMS-IRMPD approach allows the differentiation of the metabolites based on the OH and NH stretches observed, allowing the nature of the protomers and tautomers present for each metabolite to be assigned.

To assess if the fragmentation for these metabolites is specific for each protomer or tautomer, simulations of the CID mass spectra were performed using the QCxMS package (Figure 5) for each one of the species identified.^54,55^

**Figure 5:**
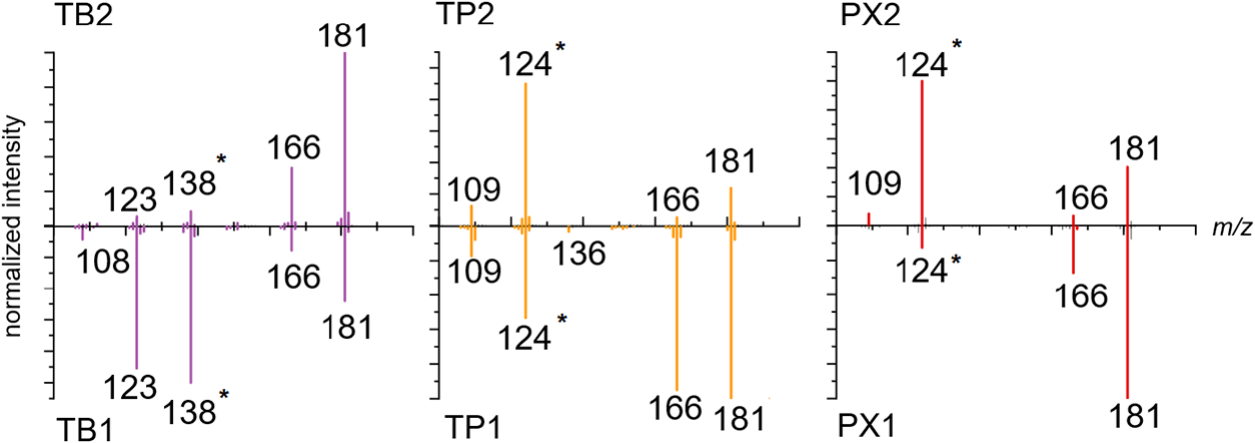
Simulated CID of metabolites with QCxMS at the GFN2-xTB level of theory for the distinct protomers of TB, TP, and PX. Fragments observed experimentally in Figure 3 are marked with *.

As previously employed for other ions, including protonated caffeine,^54^ these simulations use molecular dynamics to probe the collisions of the protonated metabolites with a collision gas, promoting their dissociation and allowing the identification of the fragments formed.

When compared to the experimental CID spectra in Figure 3, Figure 5 shows that only the fragments with *m*/*z* 138 and 124 for TB and the pair TP/PX, respectively, were observed in the CID simulation. Despite showing different intensities for the fragments generated from distinct protomers and tautomers that could, in principle, lead to their differentiation,^56^ no specific fragment ion was observed at sufficient abundance to enable their direct and unambiguous identification, highlighting the relevance of FAIMS–IRMPD for protomer and tautomer-specific differentiation of metabolites.

Figure 6 summarizes the overall FAIMS-IRMPD results and depicts the overall protomer distribution of the major caffeine metabolites analyzed in this study. It should be highlighted that protonated PX and TP show fragments with *m*/*z* 124 and, assuming the proposed fragmentation pathways shown in literature are correct,^4,43^ these fragments must differ in structure, as represented in Figure 6b,c.

**Figure 6:**
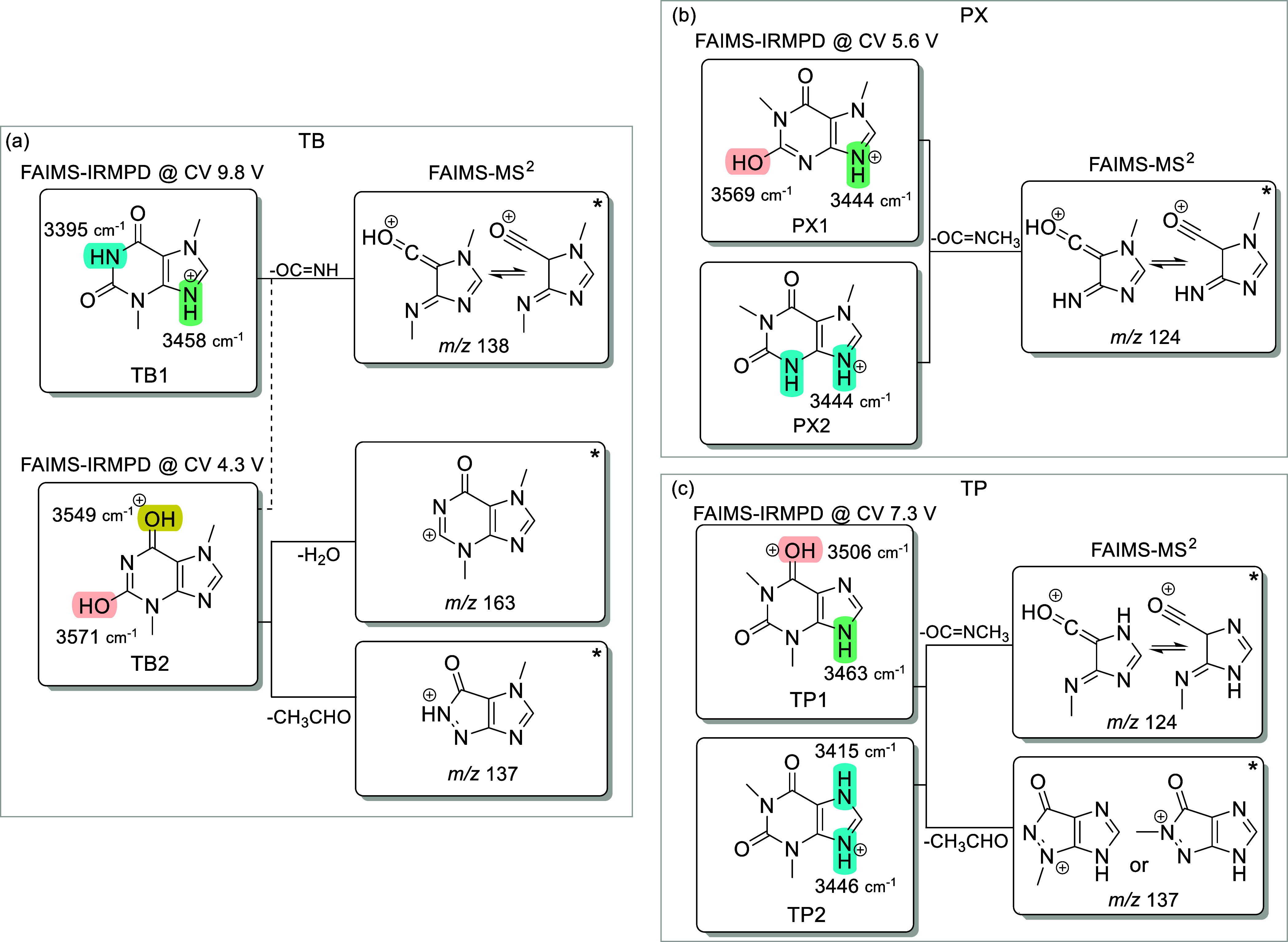
Consolidated FAIMS and IRMPD results observed for the protonated forms of the three major metabolites of caffeine: (a) theobromine (TB), (b) paraxanthine (PX), and (c) theophylline (TP). *Putative structures of the FAIMS-MS^2^ fragments are based on previous results from the literature.^4^

Although full structural elucidation of the ions with *m*/*z* 124 is beyond the scope of this work, IRMPD spectra of these fragments generated from protonated PX and TP were acquired (Figure S2a). In addition to requiring different numbers of laser pulses to reach comparable fragmentation levels, the fragments produced from each precursor display distinct spectral features, including broad absorptions in the OH and NH stretching regions at approximately 3575 and 3400 cm^–1^. These observations demonstrate that the *m*/*z* 124 fragments produced from protonated TP and PX correspond to distinct species, in agreement with previous proposals in the literature, which are reproduced in Figure 6.^4^

## Conclusions

This work evaluates the feasibility of employing the FAIMS-IRMPD scheme to evaluate protomers and tautomers of metabolites, using a mixture of the three major caffeine metabolites: theobromine (TB), paraxanthine (PX), and theophylline (TP). Our results show that using only collision-induced dissociation (CID) is not enough to identify specific protomers or tautomers of caffeine metabolites in a mixture and that, despite the excellent separation of the metabolite populations by FAIMS, even for the challenging TP/PX pair, the population-specific CID was not distinctive enough to allow unambiguous metabolite identification. Conversely, specific IRMPD spectra of the FAIMS-selected populations allow the identification of the different metabolites and their protomers and tautomers when compared to DFT-simulated absorption spectra. This analysis shows that the three major metabolites are detected as pairs of different protomers by FAIMS-IRMPD analysis, in accordance with previous results.^40,43,48^ For TB, these protomers were resolved in the FAIMS stage, while the protomers of the other metabolites were detected by ion spectroscopy.

It is worth noting that even if FAIMS-MS/MS could be used to differentiate caffeine metabolites by comparison with the CID spectra of reference standards, no tautomer/protomer-specific identification would be possible, as the simulated CID spectra for the different protomers/tautomers exhibit virtually identical fragments with only minor variations in their relative intensities.

These results highlight the capability of combining ion mobility and spectroscopy to unambiguously distinguish isomeric and protomeric/tautomeric metabolites.

## Methodology

### Sample Preparation

The standards of paraxanthine (PX) and theobromine (TB) were purchased from ChemScene and were stored at 0 °C. Theophylline (TP) was purchased from Oakwood Chemicals.

The samples were prepared in methanol (BioScie) at the following concentrations: TB 1.09 mM, TF 1.52 mM, and PX 2.05 mM. For FAIMS analysis of the isolated analytes, those samples were injected without further dilutions. When the FAIMS device was not used, the samples were diluted to achieve 0.01 mM. In all analyses, 0.01% of formic acid was added to the sample to increase ionization efficiency.

### Mass Spectrometry Analysis

The full scan mass spectrometry (MS), Collision-Induced Dissociation (CID), and Ion Mobility Spectrometry (IMS) data were acquired in positive mode by direct infusion via a nanospray ionization source using a modified Bruker AmaZon SL 3D ion trap mass spectrometer.^53,57–59^ An in-house built nanospray source was used to generate the desired ions using a borosilicate glass capillary (BF165-120-10 - Sutter Instrument Company) with a 2 μm ID tip as prepared by a Sutter Instrument Company P-2000 micropipette puller. The instrument high-voltage power supply cable was disconnected, its transfer tube was grounded, and an external HJPM-1R15 Matsusada high-voltage power supply was used to apply 1 to 3 kV to the sprayed solution via a platinum wire positioned inside the nanospray emitter.

The CID experiments were carried out under different collision energies and standard CID parameters using He as the collision gas (Air Products, 99.999%).

For the IMS analysis, a planar field asymmetric waveform ion mobility spectrometer (FAIMS, Heartland MS) with a gap width of 1.89 mm and operating at ambient pressure and temperature was coupled to the MS interface via an in-house built support. This FAIMS system was operated at a dispersion voltage (DV) of 4.5 kV (1 MHz frequency and 2:1 harmonic ratio). The compensation voltage used to filter the separated populations was scanned at 1 V min^–1^ from 2 to 12 V in relation to a positive bias voltage of 15 V to guarantee no ion rejection on the grounded inlet of the instrument. The curtain plate voltage of this device was set to 1 kV using a Bertan Associates model 313A source. The N_2_ buffer gas (99.999% purity) was generated by liquid nitrogen (White Martins) evaporation and introduced into the FAIMS cell at a 3.0 L min^–1^ flow.^60,61^

IRMPD spectra were recorded in the same MS by coupling an IR radiation beam (the 2800–3800 cm^–1^) produced by an optical parametric oscillator/amplifier (OPO/OPA) (LaserVision, ∼5 mJ/pulse, 3.7 cm^–1^ resolution) pumped by a 10 Hz Nd:YAG laser (Continuum Surelite II, 570 mJ/pulse).^53,57^ TB, TF, and PX protonated ions were irradiated with 13 to 25 laser pulses, so 50% of parent ion fragmentation was achieved.

The photofragmentation efficiency, Eff_ν̃_, was calculated as a function of the wavenumber ν̃ as Eff_ν̃_ = −ln­((*P*_ν̃_)/(*P*_ν̃_ + ∑*F*_jν̃_)), where *P*_ν̃_ represents the parent ion intensity at a given ν̃ and *F*_jν̃_ represents the *j*th fragment ion intensities at the same wavenumber ν̃. The laser intensity was monitored during the spectra acquisition as described elsewhere.^53^

To allow the acquisition of the IRMPD spectra of the protonated metabolites, the mass spectrometer Helium gas controller was set to 0.01% in order to decrease the collisional quenching of the photoexcited ions during the photofragmentation process.^62,63^ This procedure was not used for the acquisition of the IRMPD spectra of the PX and TP fragments with *m*/*z* = 124 to avoid reducing the performance of the CID step.

### Computational Methods

Gaussian 16 (Revision *C.01*)^64^ package was used to optimize the geometry and to calculate the IR absorption spectra for comparison with IRMPD spectroscopy data. The MP2/6-311++G­(d,p), ωB97X-D/6-31+G­(d,p), B3LYP/6-311++G­(3df,2pd), B3LYP/6-31G­(d), B3LYP/6-311++G­(d,p), and B3LYP/aug-cc-pVDZ levels of theory were tested to verify their suitability.^53^ In this work, the lowest RMSD comparing experimental and theoretical IR frequencies was obtained when using B3LYP/aug-cc-pVDZ level of theory and a scale factor of 0.9542. No imaginary frequencies were observed in the species reported in this work.

Simulated CID spectra were performed using the collision dissociation module implemented in the QCxMS package,^54^ with the GFN2-xTB level of theory.^55^ The default *full auto* parameters were used, apart from the collision energy, which was set to 60 eV.

## Supplementary Material



## Notes

Atomic coordinates with 3D renderings of all geometrical structures supporting IRMPD assignments, FAIMS analysis of metabolite standards, and IRMPD spectra of fragments with *m*/*z* 142 and 124 (PDF)

## Notes

This manuscript was originally submitted to *Analytical Chemistry* on August 28, 2025.
