# Supplementary material for: Infrared Ion Spectroscopy Combined with Ion Mobility Spectrometry for Identification of Caffeine Metabolite Isomers and Protomers
Source: J Am Soc Mass Spectrom. 2026 Jan 28;37(3):628–37. doi: 10.1021/jasms.5c00356 (PMC12964534; doi:10.1021/jasms.5c00356)
Supplement: Supplementary file 1 [file js5c00356_si_001.pdf]

# Infrared Ion Spectroscopy Combined with Ion Mobility Spectrometry for Identification of Caffeine Metabolite Isomers and Protomers

Gustavo Cervi and Thiago C. Correra*

Department of Fundamental Chemistry, Institute of Chemistry, University of São Paulo Av. Prof. Lineu Prestes, 748, Cidade Universitária, São Paulo, São Paulo, 05508-000, Brazil.

E-mail: tcorrera@iq.usp.br

## Supporting information

Figure S1. ..........................................................................................................2

Figure S2. ..........................................................................................................2

Coordinates for the reported species...............................................................3

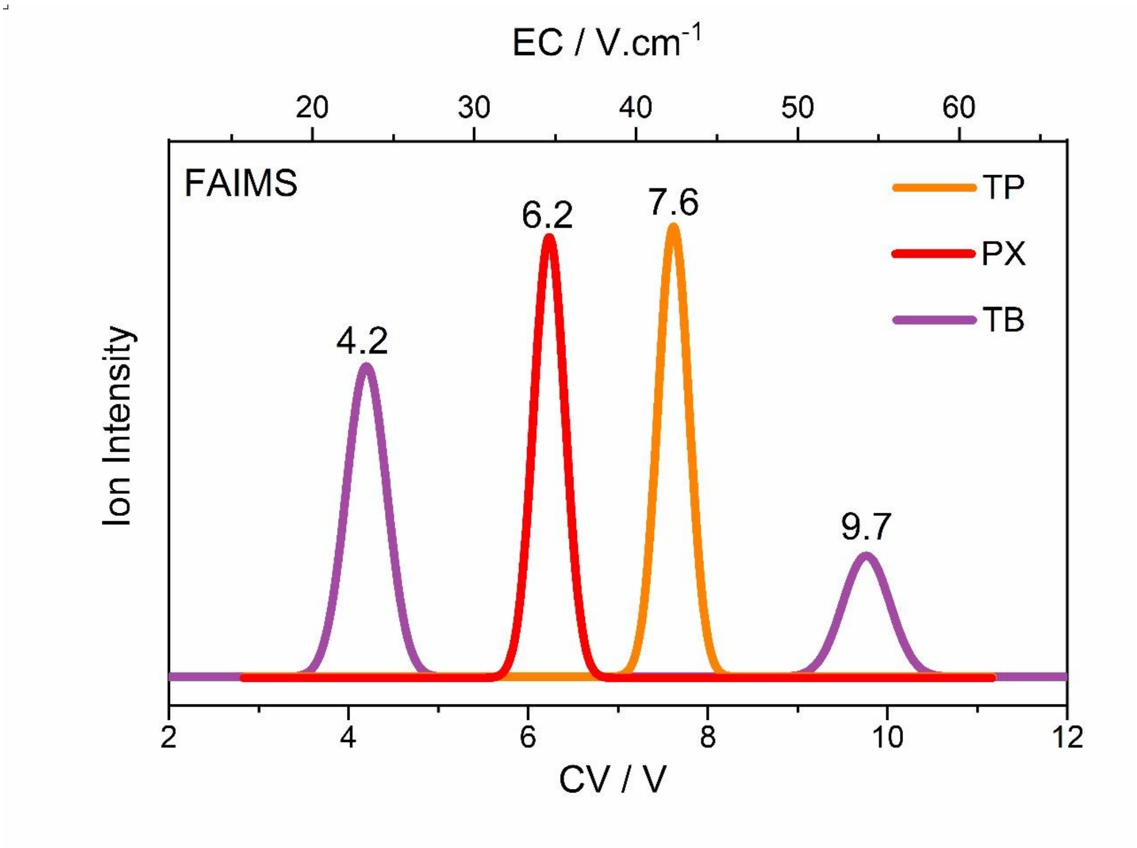


**Figure S1.** FAIMS spectrum for isolated metabolite analytical standards carried out in the same parameters used for the analysis of the mixture of metabolites reported in Figure 1.

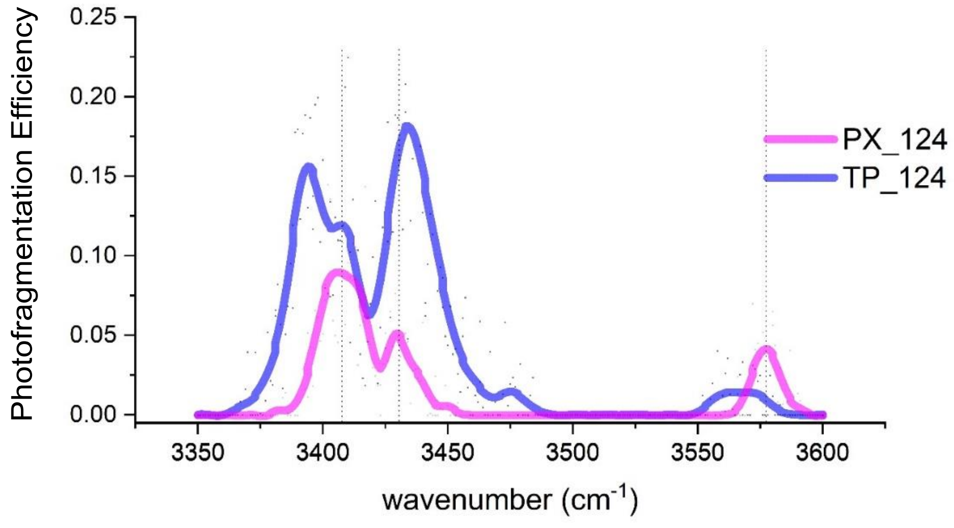


**Figure S2.** IRMPD spectra of selected fragments with *m/z* 124 from protonated PX and TP.

**Coordinates for the reported species**

PX1

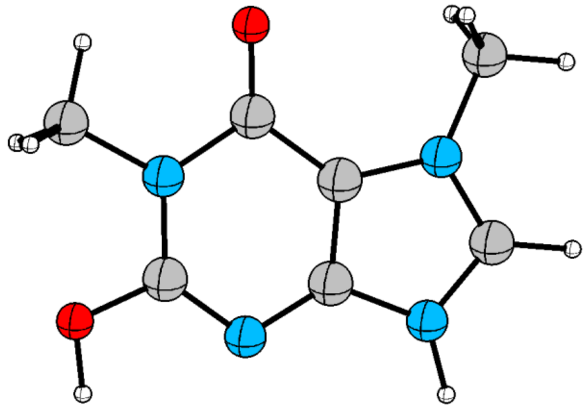


| | | | |
|---|---|---|---|
| 6 | -2.726261000 | 1.456606000 | 0.000059000 |
| 6 | 2.943601000 | 1.454821000 | 0.000234000 |
| 6 | -1.749475000 | -0.805254000 | 0.000003000 |
| 7 | -1.557319000 | 0.549463000 | 0.000100000 |
| 7 | -0.811422000 | -1.732686000 | -0.000055000 |
| 6 | -0.252574000 | 1.144962000 | 0.000046000 |
| 6 | 0.426151000 | -1.217154000 | 0.000007000 |
| 6 | 0.765341000 | 0.123133000 | 0.000173000 |
| 7 | 1.621947000 | -1.914561000 | -0.000188000 |
| 6 | 2.641974000 | -1.026105000 | -0.000175000 |
| 7 | 2.153240000 | 0.211219000 | -0.000020000 |
| 8 | -0.080543000 | 2.348748000 | -0.000110000 |
| 8 | -3.027355000 | -1.164951000 | -0.000046000 |
| 1 | -3.330784000 | 1.277119000 | -0.894871000 |
| 1 | -3.331511000 | 1.276190000 | 0.894297000 |
| 1 | -2.340502000 | 2.477154000 | 0.000707000 |
| 1 | 2.692762000 | 2.037167000 | -0.891516000 |
| 1 | 2.694074000 | 2.036003000 | 0.893124000 |
| 1 | 4.004375000 | 1.189760000 | -0.000705000 |
| 1 | 1.719803000 | -2.924370000 | -0.000423000 |
| 1 | 3.693973000 | -1.284174000 | -0.000198000 |
| 1 | -3.076661000 | -2.135330000 | -0.000115000 |

PX2

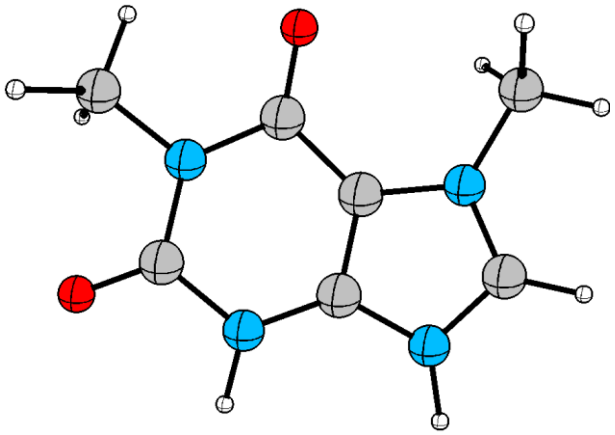

| | | | |
|---|---|---|---|
| 6 | -2.769720000 | 1.443532000 | -0.000273000 |
| 6 | 2.859087000 | 1.540033000 | -0.000241000 |
| 6 | -1.896987000 | -0.826216000 | -0.000566000 |
| 7 | -1.596567000 | 0.544071000 | -0.000090000 |
| 7 | -0.790997000 | -1.713936000 | -0.000062000 |
| 6 | -0.311310000 | 1.122905000 | 0.000668000 |
| 6 | 0.472765000 | -1.230148000 | 0.000156000 |
| 6 | 0.742270000 | 0.116340000 | 0.000241000 |
| 7 | 1.683557000 | -1.883711000 | 0.000247000 |
| 6 | 2.672064000 | -0.948089000 | -0.000070000 |
| 7 | 2.124668000 | 0.260239000 | -0.000075000 |
| 8 | -0.106677000 | 2.322998000 | -0.000215000 |
| 8 | -3.023415000 | -1.262092000 | 0.000374000 |
| 1 | -3.374750000 | 1.250243000 | -0.892045000 |
| 1 | -3.375490000 | 1.249673000 | 0.890871000 |
| 1 | -2.399426000 | 2.469269000 | 0.000224000 |
| 1 | 2.579753000 | 2.108972000 | -0.891662000 |
| 1 | 2.580246000 | 2.108931000 | 0.891362000 |
| 1 | 3.929785000 | 1.319921000 | -0.000543000 |
| 1 | -1.028406000 | -2.699165000 | 0.000499000 |
| 1 | 1.842895000 | -2.885092000 | 0.000602000 |
| 1 | 3.732489000 | -1.166789000 | -0.000219000 |

TB1

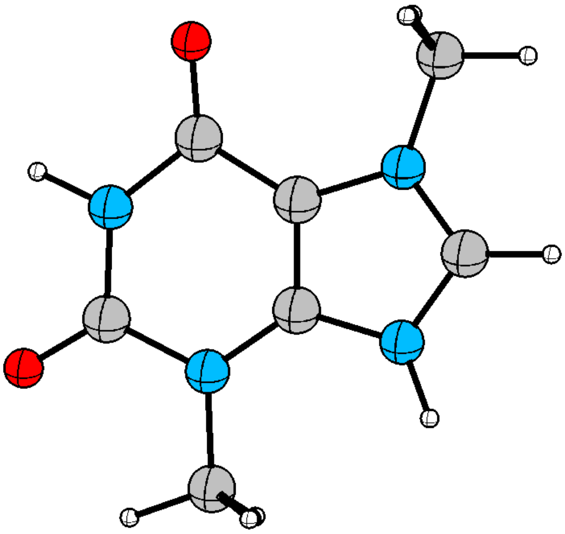

| | | | |
|---|---|---|---|
| 6 | 3.331449000 | 0.299194000 | -0.000126000 |
| 6 | -2.435671000 | -1.783979000 | 0.000145000 |
| 6 | -1.981101000 | 0.673551000 | 0.000152000 |
| 7 | -1.003008000 | 1.673640000 | 0.000127000 |
| 7 | -1.488312000 | -0.661627000 | 0.000192000 |
| 6 | 0.395706000 | 1.569690000 | 0.000204000 |
| 6 | -0.145698000 | -0.844781000 | 0.000148000 |
| 6 | 0.789158000 | 0.168632000 | 0.000327000 |
| 7 | 0.559715000 | -2.028979000 | -0.000085000 |
| 6 | 1.889203000 | -1.740008000 | -0.000310000 |
| 7 | 2.044551000 | -0.422716000 | 0.000176000 |
| 8 | 1.140182000 | 2.529243000 | -0.000057000 |
| 8 | -3.163483000 | 0.916943000 | -0.000544000 |
| 1 | 4.138667000 | -0.437826000 | -0.000431000 |
| 1 | 3.383080000 | 0.930158000 | -0.892335000 |
| 1 | 3.383478000 | 0.930355000 | 0.891884000 |
| 1 | -2.304206000 | -2.393463000 | -0.903803000 |
| 1 | -2.303073000 | -2.394340000 | 0.903326000 |
| 1 | -3.441159000 | -1.357538000 | 0.000944000 |
| 1 | -1.379433000 | 2.618711000 | -0.000222000 |
| 1 | 0.175548000 | -2.966942000 | -0.000423000 |
| 1 | 2.684611000 | -2.474635000 | -0.000242000 |

TB2

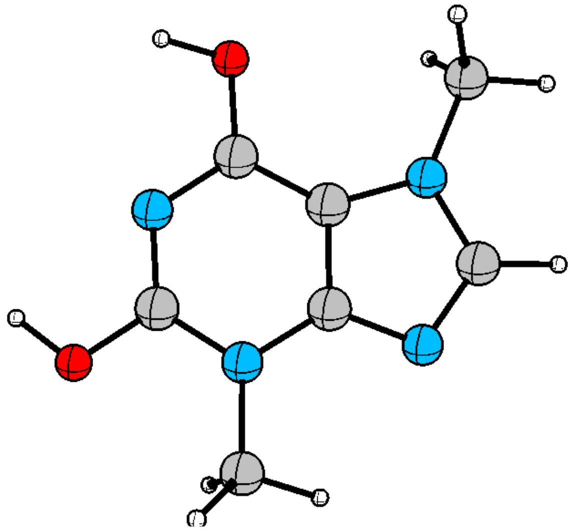

| | | | |
|---|---|---|---|
| 6 | -3.378160000 | 0.203228000 | -0.000016000 |
| 6 | 2.424730000 | -1.799902000 | 0.000148000 |
| 6 | 1.828730000 | 0.611690000 | 0.000051000 |
| 7 | 1.005351000 | 1.650052000 | 0.000013000 |
| 7 | 1.446216000 | -0.688459000 | 0.000040000 |
| 6 | -0.317590000 | 1.454079000 | -0.000043000 |
| 6 | 0.092163000 | -0.921015000 | -0.000025000 |
| 6 | -0.819111000 | 0.147109000 | -0.000068000 |
| 7 | -0.530072000 | -2.114010000 | -0.000066000 |
| 6 | -1.825166000 | -1.791743000 | -0.000169000 |
| 7 | -2.067485000 | -0.455772000 | -0.000060000 |
| 8 | -1.132151000 | 2.499494000 | 0.000023000 |
| 8 | 3.140395000 | 0.799861000 | 0.000064000 |
| 1 | -3.483935000 | 0.824335000 | 0.895942000 |
| 1 | -3.483778000 | 0.824711000 | -0.895729000 |
| 1 | -4.149220000 | -0.572377000 | -0.000250000 |
| 1 | 3.050512000 | -1.729861000 | -0.895195000 |
| 1 | 3.049858000 | -1.730275000 | 0.895988000 |
| 1 | 1.856474000 | -2.731830000 | -0.000262000 |
| 1 | -2.632958000 | -2.517326000 | -0.000222000 |
| 1 | -0.611104000 | 3.321018000 | 0.000046000 |
| 1 | 3.326552000 | 1.753414000 | 0.000226000 |

TP1

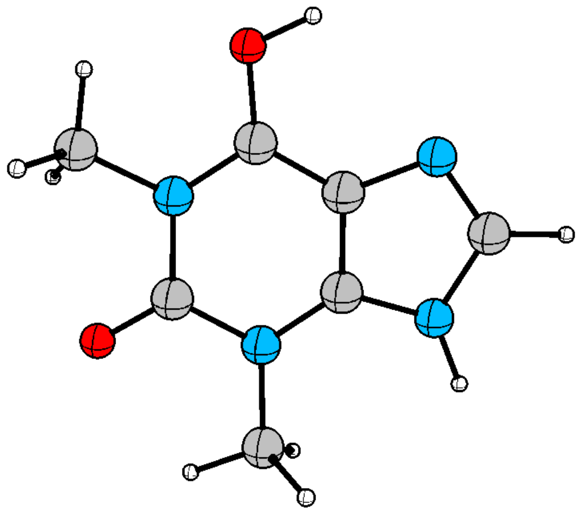

| | | | |
|---|---|---|---|
| 6 | -2.860662000 | -0.961546000 | -0.000051000 |
| 6 | 0.256731000 | 2.863376000 | 0.000038000 |
| 6 | -1.288665000 | 0.918685000 | -0.000003000 |
| 7 | -1.450466000 | -0.502753000 | -0.000084000 |
| 7 | 0.031820000 | 1.407352000 | -0.000036000 |
| 6 | -0.416962000 | -1.375083000 | -0.000037000 |
| 6 | 1.055076000 | 0.521346000 | -0.000028000 |
| 6 | 0.880284000 | -0.861156000 | -0.000026000 |
| 7 | 2.400080000 | 0.712160000 | -0.000002000 |
| 6 | 2.983644000 | -0.563512000 | 0.000030000 |
| 7 | 2.096814000 | -1.517733000 | 0.000001000 |
| 8 | -0.695696000 | -2.661299000 | 0.000003000 |
| 8 | -2.254466000 | 1.643775000 | 0.000146000 |
| 1 | -3.358082000 | -0.567460000 | 0.891447000 |
| 1 | -3.358533000 | -0.565915000 | -0.890598000 |
| 1 | -2.876350000 | -2.050818000 | -0.000977000 |
| 1 | 0.810571000 | 3.151317000 | 0.902360000 |
| 1 | 0.810906000 | 3.151336000 | -0.902070000 |
| 1 | -0.719589000 | 3.350913000 | -0.000135000 |
| 1 | 2.897257000 | 1.594782000 | 0.000017000 |
| 1 | 4.061253000 | -0.689167000 | 0.000055000 |
| 1 | 0.129446000 | -3.180623000 | 0.000007000 |

TP2

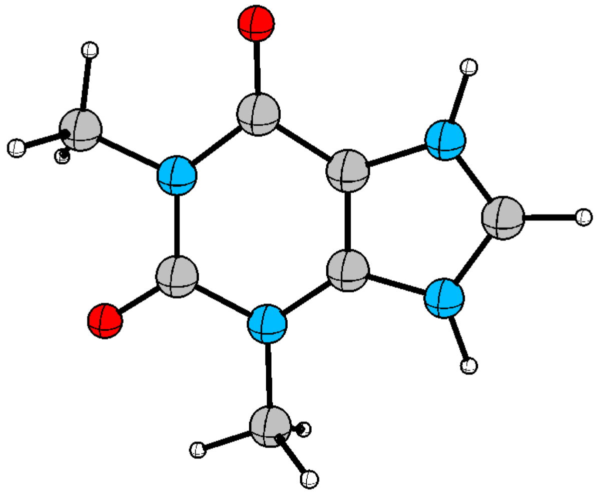

| | | | |
|---|---|---|---|
| 6 | -2.871240000 | -1.061619000 | 0.000044000 |
| 6 | 0.148496000 | 2.845027000 | -0.000122000 |
| 6 | -1.357513000 | 0.844705000 | 0.000027000 |
| 7 | -1.482066000 | -0.555804000 | -0.000011000 |
| 7 | -0.036898000 | 1.387976000 | 0.000026000 |
| 6 | -0.435770000 | -1.495963000 | -0.000045000 |
| 6 | 1.002674000 | 0.526039000 | -0.000035000 |
| 6 | 0.858326000 | -0.839799000 | -0.000101000 |
| 7 | 2.362837000 | 0.780917000 | 0.000092000 |
| 6 | 3.029619000 | -0.400959000 | 0.000100000 |
| 7 | 2.129416000 | -1.378562000 | -0.000020000 |
| 8 | -0.583752000 | -2.703741000 | -0.000050000 |
| 8 | -2.320439000 | 1.576301000 | 0.000086000 |
| 1 | -3.390917000 | -0.696668000 | 0.891786000 |
| 1 | -3.391379000 | -0.695442000 | -0.890910000 |
| 1 | -2.823790000 | -2.151379000 | -0.000681000 |
| 1 | 0.687480000 | 3.159820000 | 0.903397000 |
| 1 | 0.687787000 | 3.159581000 | -0.903536000 |
| 1 | -0.845109000 | 3.297676000 | -0.000317000 |
| 1 | 2.811430000 | 1.690517000 | 0.000030000 |
| 1 | 4.106305000 | -0.515155000 | 0.000147000 |
| 1 | 2.331149000 | -2.375703000 | -0.000014000 |
